# Changes in the Fibrinolytic System of Patients Infected with Severe Acute Respiratory Syndrome Coronavirus 2

**DOI:** 10.3390/jcm12165223

**Published:** 2023-08-10

**Authors:** Esra’a Abudouleh, Fatimah Alhamlan, Arwa A. Al-Qahtani, Marie Fe Bohol, Amal Al Hazzani, Khadija Khorfan, Morad Alkaff, Tarek Owaidah, Ahmed A. Al-Qahtani

**Affiliations:** 1 Department of Botany and Microbiology, College of Science, King Saud University, Riyadh 11451, Saudi Arabia; eabudouleh@ksu.edu.sa (E.A.); alhazzani@ksu.edu.sa (A.A.H.); 2 Department of Infection and Immunity, Research Centre, King Faisal Specialist Hospital & Research Centre, Riyadh 11211, Saudi Arabia; falhamlan@kfshrc.edu.sa (F.A.); mbohol@kfshrc.edu.sa (M.F.B.); 3 Department of Microbiology and Immunology, College of Medicine, Alfaisal University, Riyadh 11533, Saudi Arabia; 4 Department of Family Medicine, College of Medicine, Al-Imam Mohammad Ibn Saud Islamic University, Riyadh 13317, Saudi Arabia; arahalqahtani@imamu.edu.sa; 5 Department of Pathology and Laboratory Medicine, King Faisal Specialist Hospital and Research Center, Riyadh 11211, Saudi Arabia; kinda84@hotmail.com (K.K.); kaff@kfshrc.edu.sa (M.A.); 6 Department of Pathology, College of Medicine, Alfaisal University, Riyadh 11533, Saudi Arabia

**Keywords:** coagulation, coronavirus disease, thrombin activatable fibrinolysis inhibitor, plasminogen activator inhibitor, tissue plasminogen activator

## Abstract

Introduction: In this study, coagulation and fibrinolysis parameters and their association with disease severity were investigated in coronavirus disease (COVID-19) patients. Materials and Methods: COVID-19 patients (*n* = 446) admitted to our institute between 21 February 2021 and 17 March 2022, were recruited. Clinical data and staging were collected from all patients. Blood samples were collected and analyzed for several parameters of fibrinolysis and coagulation, including alpha-2-antiplasmin(α2AP) and plasminogen, thrombin activatable fibrinolysis inhibitor (TAFI), tissue plasminogen activator (tPA), plasminogen activator inhibitor-1 (PAI-1), D-dimer, and fibrinogen levels. Results: The TAFI, fibrinogen, and tPA levels were significantly higher in participants who died compared to that of patients who recovered (*p* < 0.001). However, PAI-1, tPA, and TAFI were significantly higher in patients admitted to the ICU than those of the healthy controls (*p* < 0.001 for PAI-1 and tPA; *p* = 0.0331 for TAFI). Our results showed that stage C and D COVID-19 patients had significantly higher levels of PAI-1 (*p* = 0.003). Furthermore, stage D COVID-19 patients had significantly higher tPA and TAFI values (*p* = 0.003). Conclusions: Hypofibrinolysis was the most prevalent condition among patients with severe COVID-19. In this study, several coagulation markers were elevated, making them suitable prognostic markers for hypofibrinolysis.

## 1. Introduction

Coronavirus disease 2019 (COVID-19) is a highly infectious respiratory illness caused by the severe acute respiratory syndrome coronavirus 2 (SARS-CoV-2). The first case of COVID-19 was reported in Wuhan, China in December 2019. However, the virus has spread to become a global pandemic [1]. As of January 2023, the COVID-19 pandemic has affected >120 million people worldwide, with >2.6 million deaths reported. The symptoms of COVID-19 range from mild to severe [2].

Fibrinolysis is a complex physiological process that involves the degradation of insoluble fibrin. Under normal physiological conditions, the fibrinolytic components of the body are maintained in a state of balance between pro-fibrinolytic (activation) and anti-fibrinolytic (inhibition) factors [3]. However, in certain diseases, such as respiratory diseases, cancer, and sepsis, the pathophysiology involves an altered fibrinolytic balance that leads to hypofibrinolysis (thrombosis) or hyperfibrinolysis (bleeding) [4].

A key mechanism involved in the progression of respiratory diseases is the activation of fibrinolysis. In these diseases, there is an increase in the release of fibrinolytic enzymes, such as plasmin, which break down blood clots formed in the lungs [5]. Excessive fibrinolysis can contribute to disease progression by increasing the permeability of the lung vessels, leading to fluid accumulation in the lungs and a further reduction in oxygen exchange [6].

SARS-CoV-2 can activate the fibrinolytic system, leading to excessive degradation of blood clots in infected individuals [7,8,9]. The mechanism by which SARS-CoV-2 induces fibrinolysis is not fully understood, but it is believed to involve the activation of the coagulation and fibrinolytic pathways in response to the virus [10]. The virus activates the immune system, leading to the excessive release of pro-inflammatory cytokines and increased activation of the coagulation system. This can lead to excessive activation of the fibrinolytic system. Additionally, SARS-CoV-2 infects cells that play a role in the regulation of fibrinolysis, such as blood platelets and epithelial cells of blood vessels, further contributing to the activation of fibrinolysis [11]. The virus can induce a hypercoagulable state, leading to an increase in thrombotic events and a decrease in fibrinolysis [12,13].

This report describes the correlation between the severity and progression of the disease caused by SARS-CoV-2 and changes in factors that regulate fibrinolysis. Our findings may contribute to the understanding of fibrinolytic changes and aid in the development of effective therapies.

## 2. Materials and Methods

### 2.1. Study Design and Participants

This study included 446 COVID-19 patients admitted to the King Faisal Hospital and Research Center (KFSHRC), Riyadh, Saudi Arabia between 2021 and 2022. The age of the patients ranged between 8 days and 96 years (median: 54.9 years). The percentage of children included in this study was 5.1% (*n* = 23). Diagnosis of SARS-CoV-2 was confirmed according to the World Health Organization guidelines, nasopharyngeal swabs with reverse transcriptase real-time polymerase chain reaction (RT-PCR) for SARS-CoV-2 were used to confirm the diagnosis of COVID-19 in all patients. According to our hospital’s guidelines for the management of COVID-19 patients, thromboprophylaxis (with unfractionated heparin or low-molecular-weight heparin) was administered to all adult patients and 50% of pediatric patients on the same day of hospital admission or two days later [14].

### 2.2. Blood Sample Collection

Blood samples were collected from patients hospitalized for up to 14 days on the third, seventh, and fourteenth day of admission. Blood samples were drawn into tubes containing 10 mL of ethylenediaminetetraacetic acid (EDTA), citrated blood (3.2%), and serum. All citrated samples were centrifuged within 2 h of sample collection and divided into aliquots to test for several coagulation markers at the time of hospital admission, whereas other aliquots were immediately stored at −80 °C until testing for fibrinolytic parameters.

### 2.3. Laboratory Analysis

Coagulation tests, including D-dimer, fibrinogen, prothrombin time, international normalized ratio (INR), and activated partial thromboplastin time (aPTT) were performed on the citrated samples using STAR Max^®^ (Diagnostica Stago, Marseille, France). Platelet counts from the EDTA samples was assessed using automated SYSMEX XN-10 (Sysmex Corporation, Kobe, Japan) equipment. Serum creatinine, triglyceride, and C-reactive protein (CRP) levels were measured using an automated chemistry analyzer (COBAS 601; Roche Diagnostics, Basel, Switzerland), according to the manufacturer’s instructions. An enzyme-linked immunosorbent assay with colorimetric output was performed to measure the plasma concentrations of PAI-1 (Asserachrome #00949), Activated and inactivated TAFI (Asserachrome #00616), and tPA (Asserachrome #00948). A colorimetric (STA-Stachrom) assay was used to measure plasminogen (Stachrom #00658) and α2AP (Stachrom #00659) levels, according to the manufacturer’s instructions. Normal values were determined in a control population, which comprised 40 healthy adult volunteers with no clinical evidence of viral infection and a negative RT-PCR test for COVID-19 within five days of sample collection. Patients with a history of systemic autoimmune disease, current infection, or pregnancy were excluded.

### 2.4. Clinical Data Collection

The hospital system’s electronic patient medical file was accessed to gather information about the patient’s demographics, COVID-19 stage, specifics of their symptoms, prophylaxis, and outcome throughout their stay in the hospital. All patients were clinically classified into those with disease stages A–D (Table 1). All patients with a confirmed diagnosis of COVID-19 by PCR were included. COVID-19 patients discharged after 24 h or those who were unable to provide samples for the assessment of fibrinolytic parameters were excluded.

### 2.5. Statistical Analysis

Statistical analyses were performed using GraphPad Prism Version 9. When there were two groups, skewed data were examined using the Mann–Whitney U test, while normally distributed data were analyzed using a two-sided *t*-test. One-way analysis of variance or the Kruskal–Wallis test with multiple comparison correction was used for analyses of three or more groups. The Shapiro–Wilk test was used to determine normality. Each *p*-value had two tails, and significance was defined as *p* value of < 0.05. The plasma levels of the biomarkers examined were presented as population medians, together with either the 95% confidence interval (CI) or interquartile range (IQR). A multivariate logistic regression analysis was conducted to investigate the association between several variables and the occurrence of thrombosis and the probability of death.

## 3. Results

### 3.1. Demographic and Clinical Results

COVID-19 stage C (*n* = 244, 54.6%) was the most prevalent, followed by stages B (*n* = 108, 24.3%), D (*n* = 55, 12.4%), and A (*n* = 39, 8.8%). Table 2 illustrates the demographic, clinical, and laboratory results of the COVID-19 patients.

### 3.2. Laboratory Results

Selected laboratory results for the COVID-19 patients are presented in Table 3. Markedly elevated D-dimer and fibrinogen levels were more frequently observed in ICU patients than that among non-ICU patients (D-dimer median: 1.75; IQR, 0.5 to >20 µg/mL, *p* = 0.015 and fibrinogen median: 5.04; IQR, 0.65–9.71 g/L, *p* < 0.001). It was also found that aPTT, platelet count, and INR did not differ between the ICU and non-ICU patients. In the ICU patients, CRP levels and serum creatinine were significantly higher than that of the non-ICU patients (*p* < 0.001 in both).

### 3.3. Classification of the Fibrinolytic System Based on Coagulation Markers (Hypofibrinolysis and Hyperfibrinolysis)

To assess the changes in the fibrinolytic system, we measured α2AP, plasminogen, TAFI, tPA, PAI-1, D-dimer, and fibrinogen levels in all the COVID-19 patients. No specific coagulation markers could be used to predict changes in the fibrinolytic system. A combination of these markers was used to predict changes in the fibrinolytic system. As mentioned in the literature [4,15,16,17], during hyperfibrinolysis, the activating factors of the fibrinolysis system (tPA, plasminogen, and D-dimer) increase, while the inhibitory factors (PAI-1, TAFI, α2AP, fibrinogen, and D-dimer) decrease, leading to an increase in bleeding complications.

The patients were classified as having hyperfibrinolysis, hypofibrinolysis, normal (no change in coagulation markers), or unclassified (patients that could not meet either criterion). Using a combination of seven coagulation markers, we found a prevalence of suspected hypofibrinolysis and hyperfibrinolysis of only 1.29% and 0.86%, respectively, in all the COVID-19 patient cohorts. We used new combinations of these two coagulation markers to better understand these changes. A combination of elevated levels of PAI-1and low levels of plasminogen was reported in 23.60% of the hypofibrinolysis patients, while hyperfibrinolysis was reported in 14.59% of the patients, with an increase and decrease in the α2AP and tPA levels, thereby making them more suitable candidates for predicting hyperfibrinolysis in COVID-19 patients; this is illustrated in Table 4.

### 3.4. Association between Plasma Levels of Coagulation Markers and Clinical Outcomes in COVID-19 Patients

**Figure 1:**
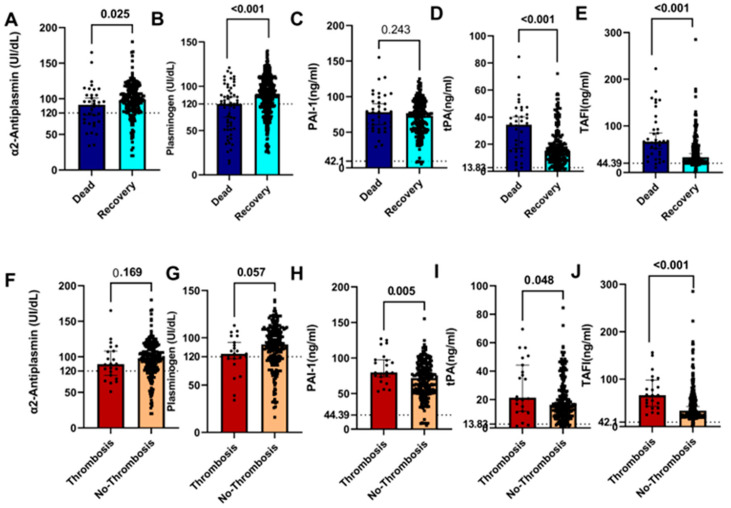
Plasma levels of different coagulation factors in COVID-19 patients. Plasma was isolated from patients who had died due to COVID-19 and those recovering from the disease (**A**–**E**). Patients with thrombosis and those without thrombosis were isolated for α2AP, plasminogen, PAI-1, tPA, and TAFI (**F**–**J**) tests. Groups were compared by using Mann–Whitney test, while normally distributed data were studied using a two-sided t-test. Levels of the coagulation factors and p values are shown in each panel. The dotted lines represent the upper limit of the normal range for the plasma level of all the coagulation markers. Data are represented as medians with 95% CI of *n* = 36 (dead patients), *n* = 197 (recovery patients), *n* = 22 (thrombosis patients), and *n* = 211 (no thrombosis).

We assessed the levels of fibrinolytic markers, such as α2AP, plasminogen, PAI-1, tPA, and TAFI, in COVID-19 patients. The plasma levels of α2AP and plasminogen were significantly higher in recovery patients than those in deceased patients (*p* = 0.025 and *p* < 0.001, respectively) (Figure 1A,B). The plasma levels of tPA and TAFI were significantly higher in the deceased patients than those of patients who recovered (*p* < 0.001) (Figure 1D,E), suggesting both parameters when elevated are likely associated with the cause of mortality. However, PAI-1 levels were similar in both patient groups (*p* = 0.243) (Figure 1C).

We assessed the levels of the aforementioned coagulation factors in patients with and without thrombosis. Our findings showed that there were no significant differences in the plasma levels of α2AP and plasminogen between the patients who developed thrombosis and those who did not (*p* = 0.169 and *p* = 0.057, respectively) (Figure 1F,G). In contrast, there were significantly higher levels of PAI-1, tPa, and TAFI in the thrombosis patients than those in patients without evidence of thrombosis (*p* = 0.005, *p* = 0.048, and *p* < 0.001, respectively) (Figure 1H–J).

### 3.5. Coagulation Biomarkers in ICU and Non-ICU COVID-19 Patients

**Figure 2:**
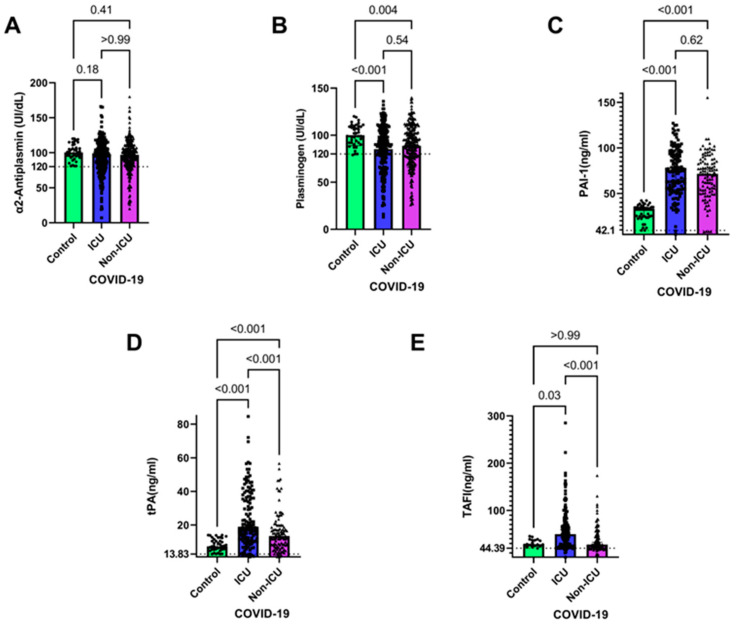
Coagulation parameters in ICU and non-ICU COVID-19 patients. Plasma isolated from blood samples of healthy individuals and COVID-19 patients either admitted or not admitted to the ICU were assessed for α2AP (**A**), plasminogen (**B**), PAI-1(**C**), tPA (**D**), and TAFI (**E**) were compared using the Kruskal–Wallis test. Levels of the aforementioned coagulation factors and p values are shown in each panel. The dotted lines represent the upper limit of the normal range for the plasma levels of all the coagulation markers. Data are represented as medians with 95% CI of *n* = 130 (ICU), *n* = 103 (non-ICU), *n* = 40 (healthy control).

Our findings revealed no significant difference in the levels of α2AP between all the patient groups. However, the plasma levels of plasminogen at 38.35% were significantly lower in the ICU and non-ICU patients than that in the healthy control group (*p* < 0.001 and *p* = 0.004, respectively) (Figure 2B). Furthermore, in comparison with the healthy control group, there was a significant increase in plasma levels of PAI-1, tPA, and TAFI in the ICU patients when compared to the healthy control group (*p* < 0.001 for PAI-1 and tPA; *p* = 0.0331 for TAFI) (Figure 2C–E). Additionally, in comparison to the healthy controls, there was a significant increase in the plasma levels of tPA and TAFI (*p* < 0.001), while the non-ICU patients had significantly lower levels of tPA and TAFI when compared with the ICU patients (*p* < 0.001), suggesting that both tPA and TAFI levels may be associated with disease severity (Figure 2D,E).

### 3.6. Association between Plasma Levels of Coagulation Markers and COVID-19 Staging

**Figure 3:**
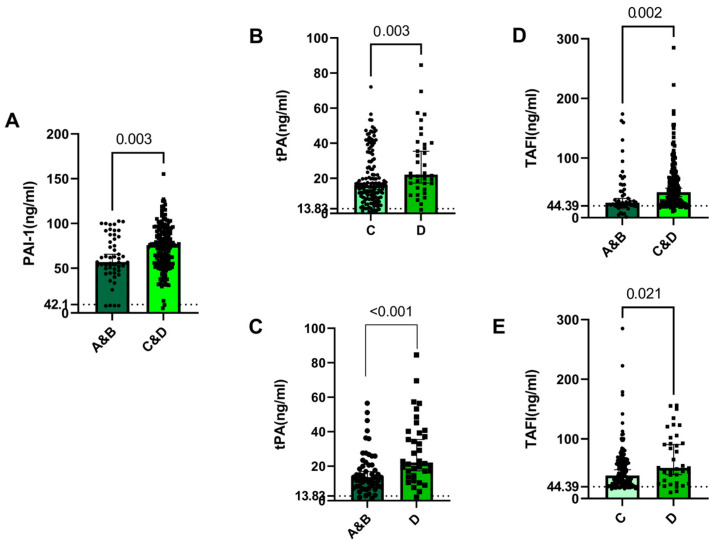
Impact of COVID-19 disease stage and plasma levels of coagulation factors in patients. Patients were grouped into four stages (**A**–**D**), and the plasma levels of PAI-1, tPA, and TAFI were compared for all the stages (**A**–**E**). Data are presented as medians with 95% CI, and data points represent individuals recruited for each group.

Our results showed that the patients in stages C and D of COVID-19 had significantly higher levels of PAI-1 and TAFI than those in stages A and B of the disease (*p* = 0.003 and *p* = 0.002, respectively) (Figure 3A,E). Additionally, the patients in stage D also had significantly higher levels of tPA and TAFI than the patients in stage C of the disease (*p* = 0.003 and *p* = 0.021, respectively) (Figure 2B,E), and significantly higher levels of tPA than the patients in stages A and B of the disease (*p* < 0.001) (Figure 3C). These data suggest an association between plasma levels of coagulation factors and disease stage.

### 3.7. Multivariate Logistic Analysis between Thrombosis and Non-Thrombosis Patients

Multivariate logistic regression analysis was conducted to investigate the association between several variables and the occurrence of thrombosis. The results showed that four of the eleven variables examined were statistically significant predictors of thrombosis. The odds of thrombosis increased by 17.4% for each unit increase in D-dimer levels (odds ratio (OR) = 1.174, 95% CI: 1.054–1.313, *p* = 0.003). Conversely, the odds of thrombosis decreased by 39.4% for each unit increase in fibrinogen level (OR = 0.6063, 95% CI: 0.3795–0.9295, *p* = 0.027). Two other variables, TAFI and PAI-1, were significant predictors of thrombosis. The odds of thrombosis increased by 1.4% for each unit increase in TAFI levels (OR = 1.014, 95% CI: 1.001–1.027, *p* = 0.032) and by 2.4% for each unit increase in PAI-1 levels (OR = 1.024, 95% CI: 1.002–1.049, *p* = 0.043) (Table S1).

### 3.8. Multivariate Logistic Analysis among Dead and Recovery

Table S2 presents the results of the multivariate logistic analysis conducted to assess the relationship between several variables and the likelihood of mortality risk. As shown in Table 2, plasminogen was negatively associated with thrombosis (OR = 0.96, 95% CI = 0.94–0.99, *p* = 0.011), indicating that higher levels of plasminogen were associated with a lower risk of dying. In contrast, TAFI (OR = 1.01, 95% CI = 1.00–1.02, *p* = 0.006) and tPA (OR = 1.03, 95% CI = 1.01–1.06, *p* = 0.006) were positively associated with dying, indicating that higher levels of these variables were associated with a higher risk of thrombosis.

## 4. Discussion

In this study, we investigated the association between coagulation markers (α2AP, plasminogen, TAFI, tPA, PAI-1, D-dimer, and fibrinogen) and various changes in the fibrinolytic system in a cohort of COVID-19 patients, as well as the severity of the disease based on disease stage and whether these patients were admitted to the ICU. The COVID-19 patients revealed changes in the levels of these markers, which may indicate changes in the fibrinolytic system [18,19].

During hyperfibrinolysis, the fibrinolysis activating factors (tPA, plasminogen, and D-dimer) increase while the inhibitory factors (PAI-1, TAFI, 2AP, and fibrinogen) decrease, increasing bleeding and associated complications [4,15,17]. While COVID-19 patients may not routinely present with either hypofibrinolysis or hyperfibrinolysis, using default criteria for predicting changes in fibrinolysis, we found a prevalence of suspected hypofibrinolysis and hyperfibrinolysis in only 1.29% and 0.86%, respectively, in all COVID-19 patients’ cohort. Hypofibrinolysis, or impaired fibrinolysis, is characterized by reduced fibrinolytic activity and increased clot stability. Elevated levels of PAI-1 and TAFI have been observed in COVID-19 patients, which can lead to reduced fibrinolysis and an increased risk of thrombotic events [19,20]. In contrast, decreased levels of tPA have been reported in COVID-19 patients, which can also contribute to impaired fibrinolysis [20]. Furthermore, low plasminogen levels were reported in 38.35% of patients admitted into the ICU and deceased patients and this was also associated with disease severity. Della et al. (2021) reported a low level of plasminogen increases the risk of mortality in COVID-19 patients [21]. On the other hand, hyperfibrinolysis, or excessive fibrinolysis, is characterized by increased fibrinolytic activity and clot breakdown. Elevated levels of D-dimer, a fibrin degradation product and marker of fibrinolysis, have been observed in COVID-19 patients, particularly in those with severe disease [19,22]. Fibrinogen is an acute-phase reactant protein, its increased level in our study indicates inflammation rather than consumption [23]. This may explain why we found an elevated level of PAI-1 and TAFI, especially in the patients that were admitted into the ICU.

On the contrary, we also reported a significant increase in the tPA levels in COVID-19 patients. These findings are also in agreement with the finding so previous studies. For example, a study by Panigada et al. (2020) found that COVID-19 patients had significantly higher levels of D-dimer, a marker of fibrinolysis, compared to healthy controls [24]. The study also found that the levels of tPA were significantly elevated in COVID-19 patients, suggesting increased activation of the fibrinolysis system. However, the levels of PAI-1, which inhibits fibrinolysis, were also elevated. Similarly, findings of elevated PAI-1 and tPA have been reported in other studies [22,25,26], indicating that the fibrinolysis system may be compromised in COVID-19 patients [25,27]. Taken together, these findings suggest that COVID-19 patients have specific changes in coagulation markers that may help us to predict changes in the fibrinolysis system and also understand the changes.

Numerous studies have reported an elevated risk of thrombotic events, particularly in severe cases that require hospitalization [28,29]. However, COVID-19 is associated with an increased risk of venous and arterial thrombosis. Pulmonary embolism has been reported in up to 31% of hospitalized COVID-19 patients, with higher rates observed in critically ill patients [30,31]. This is thought to be due to the complex interplay of factors, including inflammation, endothelial dysfunction, and alterations in coagulation and fibrinolysis [32]. In our cohort, hypofibrinolysis was observed in 22 of 446 (4.9%) COVID-19 patients, with more cases of hypofibrinolysis than hyperfibrinolysis. A similar study reported a lower incidence of thrombosis in 1.9% of COVID-19 patients [14]. However, this finding may be due to the administration of prophylactic anticoagulants earlier in the course of the disease.

In this study, arterial thrombosis was reported in 22.72% of the patients, whereas 77.27% had venous thrombosis. Deep vein thrombosis and pulmonary embolism were observed in 2/22 (9%) patients admitted to the ICU, and this was associated with COVID-19-related death. One of the children (13 years old) had a brain stroke with a high PAI-1 and low platelets. However, the patient did not receive prophylactic anticoagulants.

One trend observed in this study was a correlation between disease severity and the occurrence of thrombotic events. We found a higher incidence of thrombosis, amounting to 13/22 (59%) in males, 18 (80%) in the elderly, 15/22 (68%) in patients admitted to the ICU, and 20/22 (90%) in stages D and C COVID-19 patients. Our findings are consistent with the theory that men and older adults have a higher incidence of thrombosis [33].

All confirmed cases of thrombosis showed elevated levels of PAI-1, tPA, and TAFI, and low plasminogen levels. This is indicative of dysregulation of the fibrinolytic system, with a concomitant relationship between elevated levels of PAI-1, tPA, TAFI, and plasminogen hypofibrinolysis changes. These changes may have occurred because these patients were critically ill [30,31], suggesting that the occurrence of venous thromboembolism may be a prognostic factor for disease outcomes in critically ill patients, which can be monitored by elevated PAI-1, tPA, and TAFI levels.

Additionally, elevated levels of PAI-1 and TAFI have been reported in 88% and 45% of COVID-19 patients, respectively. This is in agreement with other studies that reported elevated plasma levels of TAFI and PAI-1, which are associated with the risk of thrombotic events [34,35]. Furthermore, low plasminogen levels were reported in 41.4% of patients admitted to the ICU and in deceased patients and were also associated with disease severity. Della et al. [21] reported that low plasminogen levels increase the risk of mortality in COVID-19 patients. Taken together, these findings suggest that COVID-19 patients have specific changes in coagulation markers that may help us predict and understand changes in the fibrinolytic system.

This study also found that the levels of coagulation markers were significantly elevated in COVID-19 patients; however, the levels of PAI-1, which inhibits fibrinolysis, were also elevated. Similarly, elevated PAI-1 and tPA levels have been reported in other studies [22,25,26], indicating that the fibrinolytic system may be compromised in COVID-19 patients [26,27].

The multivariate logistic analysis showed that D-dimer, fibrinogen, TAFI, and PAI-1 were significant predictors of thrombosis, while plasminogen and α2AP were not found to be significant predictors. These findings are consistent with previous studies that have identified D-dimer, fibrinogen, and PAI-1 as being higher in thrombosis patients [36,37].

Previous studies have shown that coagulopathy is a common finding in severe COVID-19, and that several biomarkers, such as D-dimer, fibrinogen, and platelet count, are associated with disease severity and mortality [38,39]. Our multivariate logistic analysis showed several of these biomarkers were associated with death or recovery in COVID-19 patients, further highlighting the importance of these parameters such as D-dimer, and fibrinogen levels as biomarkers of disease severity and even convalescence in COVID-19.

The results of this analysis showed that plasminogen, TAFI, and tPA were associated with mortality, which is consistent with previous studies that have shown an association between these biomarkers and COVID-19 severity and mortality [19,40]. Specifically, elevated levels of plasminogen and tPA have been shown to be associated with worse outcomes in COVID-19 patients [22], while TAFI has been shown to be a predictor of mortality in patients with sepsis [41]. This may also explain the association between TAFI and tPA levels and ICU admission, as patients with worse disease outcomes and severity are often admitted to the ICU.

Overall, these findings highlight the importance of monitoring coagulation biomarkers in COVID-19 patients, particularly plasminogen, TAFI, and tPA, which may serve as predictors of disease severity and mortality. Additionally, the results of this study suggest that fibrinogen and PAI-1 are useful biomarkers for predicting disease severity in COVID-19 patients. Further research is needed to better understand the mechanisms underlying the association between these biomarkers and COVID-19 outcomes and to determine their potential as targets for therapeutic intervention.

Finally, TAFI, PAI-1, and tPA are not readily available in hospital laboratories. While not entirely feasible, testing for TAFI, PAI-1, and tPA upon hospital admission in severe cases may be helpful in defining coagulopathy linked to COVID-19 and identifying individuals at risk of hypofibrinolysis and poorer clinical outcomes.

In summary, the multivariate logistic analysis carried out showed that COVID-19 patients are at increased risk of developing venous and arterial thromboses, which can lead to severe complications, including death. In this study, hypofibrinolysis was observed in patients with severe COVID-19, leading to a decreased ability to dissolve blood clots, which is associated with disease severity. Hyperfibrinolysis was also observed in some COVID-19 patients, but this was less prevalent. Several coagulation and fibrinolytic markers, including TAFI, PAI-1, and tPA were elevated in this study, making them suitable prognostic markers for hypofibrinolysis. Thus, understanding the mechanisms underlying thrombosis in COVID-19 patients is crucial for predicting disease progression and improving patient outcomes.

## Figures and Tables

**Table 1:** Patients to assess disease severity.

Stage	Type of Infection	Symptoms	ICU Admission
A	Asymptomatic	No symptoms	Not required
B	Mild Infection	URT symptoms Other minor symptoms (including fever and GTI)	Not required
C	Moderate Infection	Dyspnea Hypoxia Oxygen saturation <93% Absences or if pneumonia is present	Not required
D	Severe Infection	Pneumonia or either of the following: PaO_2_/FiO_2_ < 300. Respiratory rate of 30 breaths/min. >50% lung involvement on imaging within 24–48 h. Critical respiratory failure requiring mechanical ventilation, septic shock, and/or multi-organ dysfunction.	Required

URT, upper respiratory tract; GTI, gastrointestinal infections; PaO_2_, partial pressure of oxygen in arterial blood; FiO_2_, fraction of inspired oxygen; ICU, intensive care unit.

**Table 2:** Demographic and clinical data of COVID-19 patients.

Demographic Data			
**Number**		446	
Age *		55 ± 19	8D-96Y
Sex	Male	226	50.7%
	Female	220	49.3%
BMI *		28.37 ± 7.7	8.8–69.4
Clinical data			
COVID-19 Staging			
A		39	8.8%
B		108	24.3%
C		244	54.6%
D		55	12.4%
Comorbidities			
Yes		408	91.47%
No		38	8.53%
Types of comorbidities			
Bronchial asthma		29	7.11%
Cancer		55	13.49%
Hypothyroidism		36	8.82%
Heart disease		17	4.17%
Liver disease		48	11.76%
Pneumonia		34	8.33%
Renal disease		100	24.50%
Smoker		6	1.47%
Other		83	20.34%
Respiratory status			
O2 high flow		174	39%
Ventilator		94	21%
CPAP		8	1.8%
Thrombosis			
Arterial thrombosis		4	22.23%
Venous thrombosis		14	77.77%
No. of Vaccines received			
Three doses		24	5.4.%
Two doses		101	22.6%
One dose		26	5.8%
No		295	66.2%
Final outcomes			
Dead		55	12.33%
Recovery		373	83.63%
Thrombosis		18	4.04%

BMI, body mass index; CPAP, continuous positive airway pressure. * Mean ± standard deviation (range).

**Table 3:** Laboratory results of COVID-19 patients.

		All (*n* = 446)		ICU (*n* = 209)		Non-ICU (*n* = 237)		
Variable	References Range	Median	IQR	Median	IQR	Median	IQR	*p* Value
Platelets count (10^9^/L)	150–450	207	9–878	209	13–878	202	9–827	0.818
INR	0.8–1.10	1.1	0.2–21	1.1	0.8–4.3	1.1	0.90–21	0.354
PT (s)	10–14	14.9	11.6–72.6	14.9	11.60–17.38	15.1	11.80–16.90	0.498
aPTT (s)	26–40	38.75	26.3–150	38.75	26.3–150	38.75	26.30–109.6	0.597
D-dimer (µg/mL)	>0.5	1.16	0.27 to >20	1.75	0.5 to >20	1.15	0.27 to >20	**0.015**
Fibrinogen (g/L)	1.4–4.40	4.8	0.65–9.71	5.04	0.65–9.71	4.5	0.8–8.730	**<0.001**
CRP (mg/dL)	<0.9	49.1	0.3–559	69.2	0.4–559	34	0.3–292.3	**<0.001**
Serum creatinine (mg/dL)	M (0.7–1.3) F (0.6–1.1)	1	0.84–35	2.1	1.40–35	1	0.84–13	**<0.001**
Triglyceride (mg/dL)	<1.7	1.26	0.94–9.90	1.34	0.47–9.90	1.15	0.43–7.08	0.169

On the same day that plasma samples were taken, laboratory tests were performed. INR, international normalized ratio; PT, prothrombin time; aPTT, partial thromboplastin time; CRP, C-reactive protein; ICU, intensive care unit; IQR, interquartile range. Significant differences (*p* < 0.05) are in bold.

**Table 4:** Classification of the fibrinolytic system based on coagulation markers (hypofibrinolysis and hyperfibrinolysis).

Patient or Subject Classification	% Patients Showing All Coagulation Markers *		% Patients Showing PAI-1/TAFI/tPA		% Patients Showing PAI-1/PLG		% Patients Showing PAI-1/PLG/TAFI		% Patients Showing tPA/α2AP	
	*n* = 233	*p* Value	*n* = 233	*p* Value	*n* = 233	*p* Value	*n* = 233	*p* Value	*n* = 233	*p* Value
Hypofibrinolysis	1.29%	0.007	3%	0.013	23.60%	<0.001	10.73%	0.001	1.29%	0.100
Hyperfibrinolysis	0.86%	<0.001	2.15%	0.003	1.29%	0.100	1.29%	0.051	14.59%	<0.001
Normal	0.42%	<0.001	5.58%	<0.001	7.73%	<0.001	6.44%	<0.001	29.61%	<0.001
Unclassified (COVID-19 changes)	97.43	<0.001	89.27%	<0.001	67.38%	<0.001	81.55%	<0.001	54.51%	<0.001

* Coagulation markers include α2-antiplasmin (α2AP), plasminogen (PLG), thrombin activatable fibrinolysis inhibitor (TAFI), tissue plasminogen activator (tPA), plasminogen activator inhibitor-1 (PAI-1), D dimer, and fibrinogen.

## Data Availability

Data is available upon request to the corresponding author.

## Supplementary Materials

The following supporting information can be downloaded at: https://www.mdpi.com/article/10.3390/jcm12165223/s1, Table S1: Multivariate logistic analysis between thrombosis and non-thrombosis patients. Table S2: Multivariate logistic analysis between dead and recovery patients.
