# Supplementary material for: Changes in the Fibrinolytic System of Patients Infected with Severe Acute Respiratory Syndrome Coronavirus 2
Source: J Clin Med. 2023 Aug 10;12(16):5223. doi: 10.3390/jcm12165223 (PMC10455675; doi:10.3390/jcm12165223)
Supplement: Supplementary file 1 [file jcm-12-05223-s001.zip › jcm-2435663-supplementary.pdf]

Table S1. Multivariate logistic analysis between thrombosis and non-thrombosis patients.

Variable	Odds ratios	95% Confidence Interval		p value*
		Lower	Upper	
Plasminogen	1.008	0.9735	1.043	0.666
Antiplasmin	1.003	0.9754	1.03	0.848
D-dimer	1.174	1.054	1.313	**0.003**
Fibrinogen	0.6063	0.3795	0.9295	**0.027**
TAFI	1.014	1.001	1.027	**0.032**
PAI-1	1.024	1.002	1.049	**0.043**
tPA	1.024	0.9871	1.061	0.199
Platelet	1.004	0.9987	1.009	0.133
INR	0.000309	8.174E-12	2.223	0.319
PT	1.872	0.8188	7.274	0.325
PTT	1.008	0.9607	1.051	0.719

* Significant differences ($p < 0.05$) are in bold.

Table S2. Multivariate logistic analysis between dead and recovery patients.

Variable	Odds ratios	95% C.I.		p value*
		Lower	Upper	
Plasminogen	0.96	0.94	0.99	**0.011**
Antiplasmin	1.00	0.98	1.02	0.882
D-dimer	1.01	0.89	1.12	0.836
Fibrinogen	1.12	0.80	1.58	0.51
TAFI	1.01	1.00	1.02	**0.006**
PAI-1	1.01	0.99	1.03	0.442
tPA	1.03	1.01	1.06	**0.006**
Platelet	1.00	0.99	1.00	0.406
INR	0.26	0.00	6.46	0.81
PT	0.97	0.66	2.31	0.946
PTT	0.98	0.93	1.02	0.306

* Significant differences ($p < 0.05$) are in bold. .
